# Behavioral and neural adaptations in response to five weeks of balance training in older adults: a randomized controlled trial

**DOI:** 10.1186/s12952-017-0076-1

**Published:** 2017-06-13

**Authors:** Jan Ruffieux, Audrey Mouthon, Martin Keller, Michael Wälchli, Wolfgang Taube

**Affiliations:** Movement and Sport Sciences, Department of Medicine, University of Fribourg, Boulevard de Pérolles 90, 1700 Fribourg, Switzerland

**Keywords:** Aging, Postural control, Balance training, H-reflex, Transcranial magnetic stimulation

## Abstract

**Background:**

While the positive effect of balance training on age-related impairments in postural stability is well-documented, the neural correlates of such training adaptations in older adults remain poorly understood. This study therefore aimed to shed more light on neural adaptations in response to balance training in older adults.

**Methods:**

Postural stability as well as spinal reflex and cortical excitability was measured in older adults **(**65–80 years) before and after 5 weeks of balance training (*n* = 15) or habitual activity (*n* = 13). Postural stability was assessed during one- and two-legged quiet standing on a force plate (static task) and a free-swinging platform (dynamic task). The total sway path was calculated for all tasks. Additionally, the number of errors was counted for the one-legged tasks. To investigate changes in spinal reflex excitability, the H-reflex was assessed in the soleus muscle during quiet upright stance. Cortical excitability was assessed during an antero-posterior perturbation by conditioning the H-reflex with single-pulse transcranial magnetic stimulation.

**Results:**

A significant training effect in favor of the training group was found for the number of errors conducted during one-legged standing (*p* = .050 for the static and *p* = .042 for the dynamic task) but not for the sway parameters in any task. In contrast, no significant effect was found for cortical excitability (*p* = 0.703). For spinal excitability, an effect of session (*p* < .001) as well as an interaction of session and group (*p* = .009) was found; however, these effects were mainly due to a reduced excitability in the control group.

**Conclusions:**

In line with previous results, older adults’ postural stability was improved after balance training. However, these improvements were not accompanied by significant neural adaptations. Since almost identical studies in young adults found significant behavioral and neural adaptations after four weeks of training, we assume that age has an influence on the time course of such adaptations to balance training and/or the ability to transfer them from a trained to an untrained task.

**Electronic supplementary material:**

The online version of this article (doi:10.1186/s12952-017-0076-1) contains supplementary material, which is available to authorized users.

## Background

Normal aging is accompanied by structural and functional changes in the sensory and neuromuscular systems [1, 2] which lead to decreases in postural stability [3] and eventually increase the risk for falls [4]. Such age-related changes include a reduced excitability and altered modulation of spinal reflexes [5–7] and increases in corticospinal excitability [5, 6], indicating a shift towards a more cortically controlled processing of posture in older adults [8]. There is evidence that postural stability can be improved by balance training – in both young and older adults [9, 10]. However, much less is known about the neural correlates of such behavioral adaptations – especially in older adults [1].

In young adults, significant neural adaptations have been found after relatively short periods of balance training. Reductions in spinal reflex [11, 12] as well as corticospinal [12, 13] and cortical excitability [12, 14] have been reported after training periods of only four weeks. Thus, in young adults, there is evidence that improvements in postural stability after balance training can be explained by highly task-specific neural adaptations.

Since aging affects the neural control of posture, it must be assumed that it also affects the way the systems adapt to balance training. However, only very few studies investigated such effects in older adults. The available literature suggests that there actually are age-related differences in the neural adaptations to balance training. In contrast to young, older adults showed increases in spinal reflex excitability after 12 weeks of Tai Chi training [15] and alpine skiing [16]. However, the training in these two studies differed from the classical balance training used in the above-mentioned studies in young adults and lasted substantially longer. The authors of a recent study [17], which used a shorter period of six weeks of combined balance and strength training, also reported increases in spinal reflex activity and further found a decrease in corticospinal excitability after training in older adults. Thus, there is first evidence that the elderly nervous system still shows plasticity in response to balance training – already after short training periods. Penzer and colleagues [17] suggested that the increase in spinal and the decrease in corticospinal excitability after training point toward a reversion of age-related changes in postural control.

The present study aimed to shed more light on the effect of age on the neural adaptations in response to balance training. To this end, we tested a group of older adults in a number of balance and neurophysiological parameters that have previously been tested in young adults before and after a similar period of classical balance training. In particular, we investigated changes in spinal (Hoffmann’s reflex; H-reflex) and cortical excitability (H-reflex conditioning technique). The latter technique has been used in young adults who showed a decrease in the conditioned H-reflex after four weeks of balance training that was correlated to behavioral changes [12] but, to our knowledge, no training study has used this technique in older adults. Based on these previous observations, we hypothesized to find improvements in postural stability accompanied by an increase in spinal and a decrease in cortical excitability.

## Methods

### Participants

A priori calculation of the required sample size (G*Power, 3.1 [18]; 2 × 2 mixed design ANOVA, effect size f = 0.25, α = 0.05, power (1 - β) = 0.75) yielded a total sample size of 30. To compensate for possible drop outs, two additional participants were recruited for the training group. Thus, 32 older adults aged between 65 and 80 years with no known orthopedic, neurological, or other disorders were allocated to either the training or the control group using a block randomization procedure. In each group, two participants either dropped out or were excluded due to health problems emerging during the study, leaving 15 participants in the training group (age 70.1 ± 4.4 years, 8 females) and 13 in the control group (71.2 ± 5.3, 5 females). All participants were informed regarding the aim and the procedures of the study and gave their written informed consent prior to testing. The study was approved by the local ethics committee and complied with the latest Declaration of Helsinki.

### Experimental design

The design of the present study consisted of pre- and post-measurements, separated by five weeks of either balance training (training group) or habitual activity (control group). Participants of the control group were requested to maintain their usual activity pattern. During the measurements, which were carried out in the laboratory of the Movement and Sport Sciences at the University of Fribourg, behavioral as well as neurophysiological data were collected. The measurements and the training are described in detail below.

### Training

Participants of the training group attended a supervised balance training three times a week during five weeks to complete a total of 15 training sessions. The training consisted of classical one-legged balance exercises on four different unstable devices: a foam pad, a tilt board, an air-filled cushion, and a spinning top balance board. On each device, the participants performed four trials of 20 s on each leg with at least 20 s of rest between trials and five minutes between devices.

### Behavioral measures

The participants’ balance abilities were tested in a static balance task that consisted in standing still on solid ground and a more dynamic balance task that consisted in standing on a free-swinging platform. Both tasks were tested in a double- and in a single-limb condition. The one-legged tasks were performed on the right leg in all subjects. No instructions were given as for the position of the left leg. All trials were performed barefoot and with the arms akimbo. Three trials of 15 s were performed in each task and condition.

### Static balance task

The participants stood on a force plate (OR6–7, Advanced Mechanical Technology Inc., Watertown, MA, USA). They were instructed to stand as still as possible while fixating a cross on the wall 2 m in front of them. The force and torque signals of the force plate were amplified (GEN 5, Advanced Mechanical Technology Inc., Watertown, MA, USA) and recorded with custom software (Imago Record, Pfitec, Endingen, Germany) at a sampling frequency of 100 Hz. After filtering (first-order zero-lag 10 Hz low-pass Butterworth filter) the signals and converting them into physical units, the center of pressure (COP) time series on the antero-posterior as well as the medio-lateral axes were calculated and used to calculate the total COP sway path. The total COP sway path of the best of the three trials (shortest sway path) was used for statistical analysis. All processing was done in Matlab (R2015a, The MathWorks Inc., Natick, MA, USA). Since some older adults are not able to stand on one leg for 15 s and sway path can therefore not be evaluated in these participants, we counted the number of errors during one-legged stance as a second parameter. An error was defined as touching the ground with the left foot. Participants were instructed to regain the one-legged posture as quickly as possible in case they conducted an error. The average number of errors conducted during the three trials was used for statistical analysis.

In both tasks we used the best trial for the sway parameter, in order to include as many participants as possible in the analysis (all participants who performed at least one trial without error) while the rationale for using the average of the three trials for the error parameter was to avoid a big ceiling effect due to a large number of participants performing without error in their best trial.

### Dynamic balance task

For the dynamic balance task, the participants were tested on a free-swinging platform that is suspended on dampened springs (Posturomed 202, Haider Bioswing GmbH, Pullenreuth, Germany). They were instructed to reduce the oscillations of the platform and to stand as still as possible while fixating a cross on the wall 3 m in front of them. In order to record the displacements of the platform, a reflective marker was attached to the surface of the platform. The position of this marker was tracked by a motion capture system (OptiTrack, with 6 Prime 17 W cameras, NaturalPoint Inc., Corvallis, OR, USA) and recorded (120 Hz) with appropriate software (Motive:Body Version 1.7.2, NaturalPoint Inc., Corvallis, OR, USA). In Matlab, the recorded position time series in antero-posterior as well as medio-lateral direction were filtered (first-order zero-lag 5 Hz low-pass Butterworth filter) and then used to calculate the total displacement of the platform for each trial. Again, the best of the three trials was used for statistical analysis.

As for the static balance task, for the one-legged condition, the number of errors was counted for each trial. An error was defined as touching the platform with the left foot or touching the handrail that was mounted to the right of the participants. The average number of errors conducted during the three trials was used for statistical analysis.

### Neurophysiological measures

In addition to the behavioral measures, neurophysiological measurements were performed in order to assess the underlying mechanisms. With the H-reflex, the excitability of spinal reflex circuits was investigated. In order to investigate the effect of balance training on the excitability of direct (monosynaptic) corticospinal pathways, we conditioned the H-reflex with single-pulse transcranial magnetic stimulation (TMS) during perturbed stance (fast backward translations). Such a perturbation provokes different responses in the soleus muscle (SOL) that differ with respect to their latencies: a short-, a medium-, and a long-latency response (LLR). It has been shown that the latter is, at least partly, under cortical control, presumably through direct corticomotoneuronal pathways [19]. We therefore investigated the conditioned H-reflex at the time point of the LLR.

### Electromyography

In order to measure muscle responses to different stimulations (see next two sections), the activity of the SOL of the right leg was recorded with a custom electromyography (EMG) system. For this purpose, surface electrodes (Ag/AgCL; BlueSensor P, Ambu A/S, Ballerup, Denmark) were placed in a bipolar arrangement over the muscle belly. The EMG signals were amplified (200 times), band-pass filtered (10–1000 Hz), and recorded with Imago Record (4 kHz).

### H-reflex

To elicit H-reflexes in the SOL, the tibial nerve was electrically stimulated (peripheral nerve stimulation, PNS; square-wave pulse of 1 ms; Digitimer DS7A, Digitimer Ltd., Hertfordshire, UK) with the cathode (2 cm diameter) placed over the nerve in the popliteal fossa and the anode (4 × 4 cm) placed below the patella. An H-reflex recruitment curve [20] was recorded during upright standing. The maximal peak-to-peak amplitude of both M-wave (M_max_) and H-reflex (H_max_) was determined and the H_max_/M_max_ ratio was calculated in Matlab. Additionally, we calculated the root mean square of the EMG activity during the 100 ms preceding the stimulation (normalized to M_max_) in order to check for differences in background activity.

### Conditioned H-reflex

With the H-reflex conditioning technique, the excitability of corticospinal pathways can be investigated by assessing the effect of a conditioning TMS pulse on a test H-reflex. Compared to TMS alone, this more sophisticated technique allows the investigation of specific corticospinal pathways by varying the time interval (interstimulus interval, ISI) between the conditioning stimulus (single-pulse TMS) and the test stimulus (PNS) [21]. In the present study, changes in the excitability of the fastest corticospinal pathways were investigated by assessing the first observable facilitation of the H-reflex (early facilitation). It has been suggested that this early facilitation is mediated by the activation of direct monosynaptic projections from the motor cortex to the motoneuronal pool – at least for latencies no longer than 0.5–1 ms after the onset of the facilitation [21].

In a first step, the early facilitation of the SOL H-reflex was determined at rest while participants were lying in a supine position. The same setup as described above was used to record an H-reflex recruitment curve during lying. The PNS intensity for the conditioning protocol was then adjusted so that the H-reflex amplitude was approximately 50% of H_max_ and on the ascending part of the H-reflex recruitment curve. Thus we ensured that an H-reflex was visible in all trials and that there was enough tolerance to be modulated in either direction. Because of the lower H-reflex size in older adults we could not adjust the H-reflex to the 20% of M_max_ commonly used in young adults [22] as in most of the participants, this threshold was close to H_max_ or even beyond. Furthermore, the M-wave amplitude was monitored during the experiment to ensure a constant test afferent volley [23]. For the stimulation of the motor cortex, we used a butterfly coil (D-B80, MagVenture A/S, Farum, Denmark; 95 mm outer diameter, 120° angle) that was connected to a transcranial magnetic stimulator (MagPro ×100 with MagOption, MagVenture A/S, Farum, Denmark). Single pulses with a biphasic waveform were applied. By systematically moving the coil over the left motor cortex, we determined the optimal position to elicit motor evoked potentials in the SOL and fixed the coil in this position. The resting motor threshold was determined as the minimal stimulation intensity that led to a motor evoked potential of at least 50 μV in three out of five trials. This intensity was used for the conditioning pulses. Both TMS and PNS intensities were kept constant throughout the experiment.

In order to assess the early facilitation of the SOL H-reflex, ISIs between −5 and 0 ms were tested in intervals of 1 ms (negative ISIs signify that PNS was applied before TMS). Ten H-reflexes were recorded for each ISI as well as ten control H-reflexes (only PNS) for a total of 70 stimulations. The order of the stimulations was completely randomized with 4 s between two consecutive stimulations. For each ISI, the mean peak-to-peak H-reflex amplitude was expressed in percent of the mean control H-reflex amplitude. From these values, the ISI of the early facilitation was determined for each participant.

The ISI of the early facilitation and the adjacent ISIs (±1 ms) were then applied during perturbed stance. To this end, participants stood on a custom-built platform that produced fast backward translations. The stimulations were timed individually so that the peak of the H-reflex coincided with the peak of the LLR. As for the protocol applied at rest, PNS intensity was set to an intensity that elicited an H-reflex of 50% of H_max_. In cases where H-reflexes of this size were not clearly distinguishable from the background activity, PNS was set to the minimal intensity that elicited an H-reflex that was clearly distinguishable from the background activity in all trials. Thus we allowed the largest possible margin for up-modulation of the H-reflex in the conditioned trials. The TMS coil was fixed to the participants’ head with a custom-built helmet that minimized movements of the coil relative to the head. TMS intensity was the same as at rest (100% of resting motor threshold). Ten H-reflexes for each ISI as well as 10 control H-reflexes were recorded in a randomized order with an interval of 5 to 10 s between trials. The amount of the early facilitation of the H-reflex was calculated as described above. To ensure similar levels of background muscle activity in both groups and sessions, the EMG signal during the 100 ms preceding the perturbation was analyzed (root mean square of the EMG signal normalized to M_max_).

### Statistical analysis

All output variables were checked for normal distribution prior to analysis (Kolmogorov–Smirnov test). Data sets that significantly differed from a normal distribution were logarithmically transformed (indicated in the respective results section). Two-way mixed design analyses of variance with the factors session (pre vs. post) and group (training vs. control) were performed on each dependent variable separately. Significant effects were followed up by Bonferroni-corrected post hoc Student’s *t*-tests.

The number of errors during one-legged stance in both tasks could not be transformed into a normal distribution because there were too many participants performing without errors. Thus, these data were analyzed using non-parametric tests: group differences were analyzed with a Mann-Whitney test on the individual differences between the two sessions ([number of errors at post-measurement] – [number of errors at pre-measurement]).

The alpha level was set at .05 for all tests. Effect sizes are reported in form of Pearson’s correlation coefficient *r*, where *r* = .1, *r* = .3, and *r* = .5 denote a small, medium, and large effect, respectively [24]. All statistical analyses were performed using SPSS Statistics 23 (IBM Corporation, Armonk, NY, USA).

## Results

**Fig. 1:**
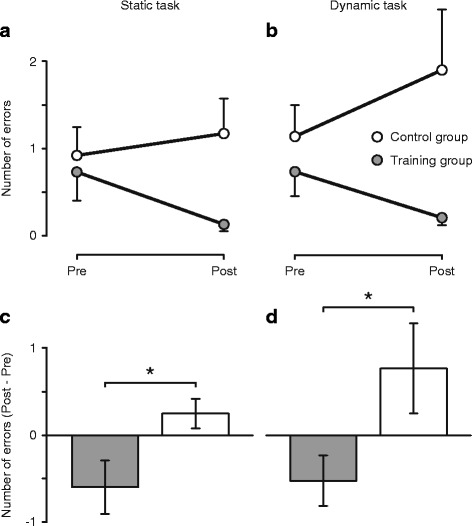
Balance training reduced the number of errors during one-legged stance. Mean number of errors committed during 15 s of one-legged stance on stable ground (**a**; Static task) and on a free-swinging platform (**b**; Dynamic task) before (Pre) and after (Post) five weeks of balance training (Training group, filled circles) or habitual activity (Control group, open circles). In (**c**) and (**d**), the change from pre to post is displayed for the static and the dynamic task, respectively. An error was defined as touching the ground with the foot of the non-supporting leg or holding on to a handrail. For each participant, the mean of three trials was used. *The change from pre to post differed significantly between groups (*p* < .05). *n* = 15 for the training group, *n* = 13 for the control group for (**a**) and (**c**); *n* = 12 for the training group, *n* = 13 for the control group for (**b**) and (**d**). Error bars represent the standard error of the mean

For different reasons (e.g., not able to perform particular balance task, no H-reflex could be elicited, measurement problems), some participants had to be excluded from individual analyses. The number of participants in each group that was included in the analysis is specified for each output parameter in Tables 1 and 2 and Fig. 1. No group differences were found for age or sex ratio for any analysis. Due to the large number of output parameters, only significant results are presented in detail. The datasets generated and analyzed in the current study are available as electronic supplementary material.

**Table 1:** Group results for the sway parameters

		Static balance task		Dynamic balance task	
		Two-legged	One-legged	Two-legged^a^	One-legged
Group	Session	COP sway (cm)		Sway (cm)	
Training group	Pre	19.0 ± 6.4	108.5 ± 45.9	0.9 ± 0.4	6.6 ± 3.1
(*n* = 15)	Post	19.9 ± 5.6	93.4 ± 36.2	1.2 ± 0.6	6.6 ± 3.1
Control group	Pre	22.8 ± 8.8	87.3 ± 19.9	1.4 ± 0.5	9.2 ± 8.8
(*n* = 13)	Post	21.4 ± 7.4	97.8 ± 42.6	2.0 ± 1.3	9.3 ± 8.3
*n* (TG/CG)		15/13	11/6	14/12	9/7

^a^Significant main effects of group (*p* = .013) and session (*p* = .008)

Group mean values ± SD before (Pre) and after (Post) five weeks of balance training (training group, TG) or habitual activity (control group, CG). The static and the dynamic balance tasks consisted in standing still on stable ground or standing on a free-swinging platform, respectively. Sway values represent the total displacement of the center of pressure (COP) or the platform, respectively, during a 15 s trial (best of three trials). *n* (TG/CG) = number of participants in each group included in the respective analysis

**Table 2:** Group results for the neurophysiological parameters

		H-reflex^a^	Conditioned H-reflex	
		Ratio H_max_/M_max_	ISI	Facilitation
Training group	Pre	0.36 ± 0.16	−3.8 ± 0.7	22 ± 16%
(*n* = 15)	Post	0.32 ± 0.17		21 ± 13%
Control group	Pre	0.46 ± 0.18^b^	−3.8 ± 0.4	11 ± 12%
(*n* = 13)	Post	0.32 ± 0.16^b^		14 ± 14%
*n* (TG/CG)		14/9	9/6	

^a^Significant main effect of session (*p* < .001) and interaction between session and group (*p* = .012). ^b^Significant reduction from pre to post (*p* < .001)

Group mean values ± SD before (Pre) and after (Post) five weeks of balance training (training group, TG) or habitual activity (control group, CG). The ratio H_max_/M_max_ represents the ratio between the maximal H-reflex and M-wave amplitudes. Values for the conditioned H-reflex represent the amount (in % of the control H-reflex) and the corresponding interstimulus interval (ISI, in ms) of the early facilitation of the H-reflex. Both parameters were measured in the soleus muscle. *n* (TG/CG) = number of participants in each group included in the respective analysis

### Behavioral measures

Behavioral data are presented in Table 1 and Fig. 1. Training had no significant effect on the COP sway path during one- and two-legged stance in the static balance task. Sway data of the dynamic task were logarithmically transformed because of non-normality. For the two-legged task, significant main effects of group, *F* (1, 24) = 7.23, *p* = .013 and session, *F* (1, 24) = 8.22, *p* = .008, were found but there was no interaction of the two factors. The group effect is due to a longer sway path in the control group compared to the training group while the session effect is due to a longer sway path during the post-measurement compared to the pre-measurement (see Table 1). No effect was found for the sway path in the one-legged condition of the dynamic task.

However, the Mann-Whitney tests indicated that training had a significant effect on the number of errors during one-legged stance in both the static, *U* = 60.00, *p* = .050, *r* = −.36, and the dynamic task, *U* = 41.00, *p* = .042, *r* = −.42. These effects are due to the training group committing fewer and the control group committing more errors on average at the post-measurement (see Fig 1). Moreover, the improvements of the training groups are likely to be underestimated because of ceiling effects: nine out of 15 and five out of 12 participants, respectively for the two tasks, performed without any error during the pre-measurement and thus could not further improve in this output parameter. Additionally, the remaining three participants of the training group, who could not be included in the analysis of the dynamic task, were not able to perform the task before the training but performed without error after it.

### Neurophysiological measures

#### H-reflex

Background EMG analysis showed that background muscle activity was similar across groups and sessions. H_max_/M_max_ ratios showed a significant effect of session, *F* (1, 23) = 27.77, *p* < .001, as well as a significant interaction of session and group, *F* (1, 23) = 8.02, *p* = .009. Post hoc tests revealed that these effects were mainly due to the control group which showed significantly reduced spinal reflex excitability during the post-session, *t* (10) = 4.90, *p* < .001, *r* = .84, while there was no significant time effect in the training group, *t* (13) = 2.02, *p* = .065, *r* = .49 (see Table 2).

#### Conditioned H-reflex

During lying, all participants exhibited constant M-wave amplitudes throughout the experiment suggesting that a constant number of nerve fibers was excited by the stimuli [23]. The ISI of the early facilitation was, on average, −3.8 ± 0.6 ms and did not differ between groups. For the perturbation protocol, no differences in background EMG were found between groups or sessions, suggesting similar levels of background activity. For the early facilitation of the H-reflex during the perturbation, no significant main effect of group, *F* (1, 13) = 2.92, *p* = 0.111, or training, *F* (1, 13) = 0.56, *p* = 0.816, nor a significant interaction, *F* (1, 13) = 0.15, *p* = 0.703, was found (see Table 2).

## Discussion

Our results showed a training effect on the number of errors during the more challenging one-legged tasks. This effect is probably still underestimated as participants of the training group generally committed very few or no errors at all already at the pre-measurement and thus had very little or no room for improvements. No significant training effects were found for the sway path during both the one- and the two-legged tasks. This is in contrast to the results found in young adults, where the sway path was reduced after four weeks of balance training – at least for the one-legged stance [11, 12]. For the two-legged tasks, we assume that they were too easy so that participants already showed minimal sway paths which could not further be reduced by training. This is in line with the results of Penzer and colleagues [17] who found, in older adults, only slight improvements in bipedal stance after six weeks of balance training and only in an unstable condition (standing on foam). A potential factor explaining the absence of improvements in postural sway during the one-legged tasks in the present study, besides the low number of participants who could be included in the analysis, is the discrepancy between the tasks trained and those tested. The lack of transfer from the trained tasks (standing on unstable devices) to the tested tasks (standing on stable ground or standing on the Posturomed) could have masked a training effect. This seems even more likely when taking into account the personal observations we made during the training sessions that most of the participants clearly improved in the tasks they trained. Besides that, a recent meta-analysis on effects of balance training in older adults has shown that the effects generally increase with increasing volume and that interventions shorter than 11 weeks have rather low effects on balance performance [25]. This suggests that for older adults, the training period of five weeks was probably too short to induce meaningful adaptations. A longer training period seems to be crucial, particularly with regard to the ability to transfer training effects to untrained tasks.

We found no significant training-related adaptations in the neural parameters. Spinal reflex excitability data showed an effect of session as well as an interaction of session and group, however, these effects were due to the control group showing a decrease in H-reflex amplitude. Thus, the interaction effect can hardly be interpreted as a training-related adaptation. We cannot plausibly explain these fast changes in the control group but it should be noticed that they were accompanied by behavioral alterations (increased number of errors during one-legged stance). In two previous studies that found an increase of spinal excitability after training in older adults [15, 16], the interventions were different (Tai Chi and alpine skiing, respectively) and substantially longer (12 weeks). Furthermore, in one of the studies [16], adaptations were only found during a dynamic task but not during standing on solid ground nor at rest. In another study, Penzer and colleagues [17] found adaptations in spinal excitability after six weeks of balance training. However, they found no changes in H_max_ but rather in the slope of the input-output relation. This indicates that perhaps more sensitive parameters than H_max_ must be investigated in order to detect subtle neural adaptions to balance trainings of a relatively short duration in older participants. Studies in young adults, on the other hand, consistently showed reduced H-reflex sizes after four weeks of balance training [11, 12]. Also cortical excitability – measured by conditioning the H-reflex with single-pulse TMS – was not significantly changed after training in the present study. Interestingly, an almost identical test protocol was used in young adults and revealed a large decrease in cortical excitability after four weeks of balance training [12].

We concluded above that the training period of five weeks was probably too short to induce large behavioral adaptations in the older participants of the present study – notably in transfer tasks. Since neural adaptions form the basis for behavioral improvements, we assume that the above conclusion is also – or particularly – true for neural adaptations. The absence of such changes could explain why no improvements could be found in the sway parameters. As for behavioral measures, neural adaptations were not investigated in the dynamic one-legged tasks that were actually trained but rather in two-legged transfer tasks as it had previously been done in young adults: during upright stance on solid ground (spinal reflex excitability) and during a perturbation (cortical excitability). Since older adults showed no significant neural adaptations in the present study, as opposed to young adults who showed large adaptations in previous studies with almost identical testing and training, there seem to be age-related differences in the neural plasticity induced by balance training. If older adults actually do show adaptations in these neural parameters, it seems reasonable to assume that they occur at a slower rate than in young and/or that older adults are less able to transfer adaptations from a trained to an untrained balance task.

A last limiting factor that needs to be discussed is the sample sizes. For different reasons, the actual sample sizes that were included in the statistical analyses were for some parameters considerably smaller than the required sample size that had been calculated a priori (see results section). The statistical tests of the concerned parameters might therefore be underpowered.

## Conclusions

The present study investigated the effect of age on the behavioral and neural adaptations in response to balance training. Contrary to our expectations, we found no significant neural adaptations after five weeks of training in older adults. Accordingly, participants did not improve in parameters of postural sway. Since almost identical studies in young adults found significant behavioral as well as neural adaptations after four weeks of training, we assume that age has an influence on the time course of such adaptations to balance training and/or the ability to transfer them from a trained to an untrained balance task. However, there are indications, from the present and previous studies, that balance training improves older adults’ postural control. We therefore need more and especially longer training studies with intermediate measurements – perhaps also investigating different parameters and/or using different methodologies – in order to get a conclusive picture of the (time course of) neural mechanisms underlying behavioral adaptations to balance training in older adults.

## Additional file

Additional file 1: Complete data set. (XLSX 51 kb)

## Abbreviations

COP: Center of pressure
EMG: Electromyography
H_max_: Maximal H-reflex amplitude
H-reflex: Hoffmann’s reflex
ISI: Interstimulus interval
LLR: Long-latency response
M_max_: Maximal M-wave amplitude
PNS: Peripheral nerve stimulation
SOL: M. soleus
TMS: Transcranial magnetic stimulation
